# Current and evolving treatment strategies in adult immune thrombocytopenia

**DOI:** 10.1007/s12254-018-0428-7

**Published:** 2018-08-15

**Authors:** Jan-Paul Bohn, Michael Steurer

**Affiliations:** Department of Internal Medicine V, Medical University of Innsbruck, Anichstraße 35, 6020 Innsbruck, Austria

**Keywords:** Dexamethasone, Rituximab, Romiplostim, Eltrombopag, Splenectomy

## Abstract

Immune thrombocytopenia (ITP) is an acquired autoimmune phenomenon resulting in low platelet count and increased bleeding risk. Goals of upfront management include prompt control of severe bleeding—which is rare—as well as induction and maintenance of a hemostatic platelet count. Thus, optimal management of ITP patients is often challenging and requires a highly individualized approach. Many patients may not suffer significant bleeding despite severe thrombocytopenia and the risk of toxicity associated with treatment may outweigh its benefit. Most patients treated with standard first-line regimen of glucocorticoids achieve an initial response. However, the rate of long-term remission remains low and multiple lines of therapy are often required. Current investigations aim at defining the subgroup of patients at risk of relapse and providing intensified risk-balanced induction regimens to improve long-term disease control.

This short review summarizes current and emerging treatment strategies in adult ITP.

Immune thrombocytopenia (ITP) is an uncommon but distinct autoimmune-mediated condition defined by a platelet count <100,000/µL due to accelerated platelet destruction and impaired thrombopoiesis. The incidence of ITP is estimated to be between 1.9 and 6.4/100,000/year in children and approximately 3.3/100,000/year in adults with a slight overall female predominance [1]. Its clinical course is characterized by a variable bleeding tendency and disturbed health-related quality of life (QoL). Front-line management aims at rapid control of severe bleeding and its prevention by providing hemostatic platelet counts. However, many patients with ITP do not experience significant bleeding despite severe thrombocytopenia.

Accordingly, the decision to treat ITP patients can be challenging because the risk of bleeding is difficult to estimate. There is no robust risk model predicting bleeding complications and patients may more likely experience treatment-related toxicities than severe bleeding [2, 3].

This short review aims to summarize current and evolving treatment strategies and discuss clinically relevant obstacles to overcome.

## Platelet count and bleeding risk

Although more severe thrombocytopenia (e. g., platelet count <10,000/µL) is associated with a higher risk of bleeding, the degree of thrombocytopenia does not strictly correlate with bleeding symptoms.

Even at platelet counts <30,000/µL the link between platelet count and bleeding risk is weak. Bleeding events are the result of various risk factors and remain unpredictable with currently available laboratory analysis as they may also occur at higher platelet counts [2, 3]. Consequently, ITP is a very heterogeneous disease. Lacking an evidence-based threshold for what constitutes a safe platelet count, the American Society of Hematology recommends treatment for patients with a platelet count <30,000/µL even in the absence of significant bleeding symptoms. Other guidelines merely recommend not to treat at platelet counts >50,000/µL [4]. Most patients with stable platelet counts >30,000/µL and no significant bleeds do not require ITP-specific treatment. The risk of toxicity associated with treatment may outweigh its benefit in otherwise asymptomatic patients with mild to moderate thrombocytopenia and a very low bleeding tendency. Thus, the occurrence of bleeding symptoms and the overall background bleeding risk are critical determinants for treatment decisions requiring a highly individualized approach rather than strict platelet thresholds for the initiation of treatment.

## Fatigue and health-related QoL

Platelet count alone does not adequately reflect the natural history of ITP and response to treatment. Although incompletely understood, fatigue in patients with ITP has been shown to be worse compared to the general population across several studies [5]. Multiple factors may contribute, such as immune alterations, fear of bleeding, restriction in daily activity, comorbidities as well as treatment-related side effects [6]. As such, health-related QoL, strongly affected by fatigue, can serve as an additional tool for evaluation of daily well-being. It has been demonstrated that the health-related QoL of ITP patients is not only worse than that of the general U. S. population but also of patients with arterial hypertension, arthritis, or cancer [7]. Whereas early ITP studies used rather general QoL questionnaires (e. g., EQ-5D, SF-36) a more disease-specific tool, the 50-item ITP Patient Assessment Questionnaire (ITP-PAQ) was successfully introduced, which can be applied to describe QoL changes over time in a treatment group [8]. Nevertheless, the effect of medications on QoL is understudied, and greater emphasis should be given to this topic in future trials.

## First-line treatment

Oral corticosteroids (prednisolone 1–2 mg/kg per day over 2–4 weeks with a 4–6 week taper) have traditionally been the standard first-line treatment in adults with initial response rates between 60 and 80%. However, relapses after drug tapering are frequent, occurring in 70–80% of patients [9].

As a result, investigators are currently evaluating whether intensification of up-front therapy with high-dose dexamethasone and rituximab may enhance long-term outcome.

Emerging data favors shorter courses of high-dose dexamethasone (40 mg orally per day for 4 consecutive days with no taper in repeated cycles every 2–4 weeks) compared to prednisolone.

In a meta-analysis of nine randomized trials (1138 patients) evaluating outcomes for different glucocorticoid regimens, dexamethasone was associated with a higher overall response at 2 weeks (79 versus 59%), deeper response at 2 weeks (complete response 64 versus 36%), and fewer bleeding manifestations within the first 10 days (12 versus 24%). Notably, dexamethasone was associated with fewer toxicities (24 versus 46 adverse events per 100 patients) [10].

Long-term response rate analysed at 6 months may not be enhanced by one or two cycles of high-dose dexamethasone compared to prednisolone (Table 1; [11, 12]). Early evidence, however, suggests superiority when given for at least three cycles [13].

**Table 1:** Platelet count responses to first-line treatment with high-dose dexamethasone in selected randomized controlled trials in adults with newly diagnosed immune thrombocytopenia (ITP)

Trial	Regimen	*n*	Median time to response		CRR at 3 months		ORR at 6 months	
Wei et al. [11]	Dexamethasone 40 mg/day d1–4 × 1–2 cycles	95	3 months	*p* < 0.01	48%	*p* < 0.01	40%	*p* > 0.05
	Prednisone 1 mg/kg/day × 4 weeks	97	6 months		26%		41%	
Bae et al. [12]	Dexamethasone 40 mg/day d1–4 × 1 cycle	76	–		–		25%	*p* > 0.05
	Prednisone 1 mg/kg/day × 4 weeks	75	–		–		36%	

*d* day, *CRR* complete response rate defined as platelet count ≥100,000/µL, *ORR* overall response rate defined as platelet count ≥30,000/µL

Rituximab is an anti-CD20 monoclonal antibody approved for malignant B‑cell lymphomas and used in numerous autoimmune conditions [14]. Typical dosing in ITP is adopted from lymphoma treatments (4 infusions in weekly intervals at 375 mg/m^2^). Lower fixed doses (e. g., 100 mg) may also be sufficient to achieve a platelet count response, but ideal dosing in patients with ITP remains unclear [15].

Two randomized trials have evaluated the addition of rituximab to high-dose dexamethasone in patients with newly diagnosed ITP (236 patients). Response rates at 6 (63 vs 36%, *n* = 103 and 58 vs. 37%, *n* = 133) and 12 months (53 vs. 33%; *n* = 133) were significantly enhanced in the combination therapy arms at the cost of more frequent grade 3 and 4 adverse events (Table 2). As such, longer follow-up is needed to determine whether greater long-term response justifies greater toxicity [16, 17]. Combination therapy with high-dose dexamethasone and rituximab is currently not recommended in newly diagnosed ITP outside of a clinical trial.

**Table 2:** Response to first-line treatment with high-dose dexamethasone and rituximab in selected randomized controlled trials in adults with newly diagnosed immune thrombocytopenia (ITP)

Trial	Regimen	*n*	ORR at 6 months		Grade 3/4 adverse events	
Zaja et al. [15]	Dexamethasone 40 mg/day d1–4 × 1 cycle plus rituximab 375 mg/m^2^ weekly × 4	49	63%	*p* = 0.04	10%	*p* = 0.08
	Dexamethasone 40 mg/day d1–4 × 1 cycle	52	36%		2%	
Gudbrandsdottir et al. [16]	Dexamethasone 40 mg/day d1–4 ≤ 6 cycles plus rituximab 375 mg/m^2^ weekly × 4	62	58%	*p* = 0.02	26%	*p* = 0.04
	Dexamethasone 40 mg/d d1–4 ≤ 6 cycles	71	37%		11%	

*d* day, *ORR* overall response rate defined as platelet count ≥50,000/µL

Intravenous immunoglobulin (IVIg) is typically administered at 1 g/kg for one to two days, depending on when a response occurs. Its effect is generally transient but raises the platelet count more rapidly than glucocorticoids within 24 to 48 h [18]. IVIg is usually reserved for patients with severe bleeding, as preparation for patients in need for an urgent invasive procedure and/or bridging to more definitive second-line treatment in patients refractory to glucocorticoids. As a result, it is considered more of a rescue option in ITP than routine therapy.

Whether first-line treatment enhances health-related QoL remains to be elucidated in the absence of randomized controlled clinical trials addressing this issue.

## Second-line treatment

As far as second-line therapy is concerned, splenectomy has been the treatment of choice for the last six decades. Two out of three patients achieve remission after splenectomy and long-term response rate is approximately 60% at 5–10 years [19]. Recently, however, the advent of rituximab and thrombopoietin receptor agonists (TRA) led to a decline in splenectomy [20]. Whether efficacy and safety of these newer agents is comparable with splenectomy is currently subject to investigation.

Efficacy profile seems favorable for a single course of rituximab with initial overall response and complete response rates of 55–75% and 40–60%, respectively. However, this may not be followed by long-term remission given inferior sustained response rates of 20–40% at 2 years, and about 20% at 5 years [21].

So far, rituximab does not appear to improve health-related QoL as measured by ITP-PAQ or SF-36 [22].

TRA stimulate the production of megakaryocytes and ultimately platelets by binding to and activating the thrombopoietin receptor. Two TRA, romiplostim and eltrombopag, are currently licensed for adults with chronic ITP and achieve a remission in over 80% of patients [23].

Dosing of romiplostim ranges from 1 to 10 µg/kg administered as a once weekly subcutaneous injection and adjusted to maintain a platelet count >50,000/µL.

Eltrombopag is given as a once-daily pill at varying doses (typically 50 mg) to establish a platelet count of >50,000/µL.

When given continuously, over 90% of responding patients maintain their responses, typically at a stable dose (Table 3) [24, 25]. Single case series also highlight that a significant proportion of patients may experience sustained responses despite treatment discontinuation after 1 or 2 years [26, 27]. Cross-resistance has yet not been observed, meaning that patients unresponsive to one agent may benefit from the other [28].

**Table 3:** Platelet count response to thrombopoietin receptor agonists in selected patients with chronic ITP

Study	Regimen	*n*	Median time to response	ORR	CRR	Sustained ORR^a^	Median time of response
Gonzalez-Lopez et al. [24]	Eltrombopag 50 mg daily	164	12 days	89%	80%	75% at 15 months	–
Mazza et al. [25]	Romiplostim 1–10 µg/kg s. c. weekly	55	4 weeks	80%	44%	–	30 months^c^
	Eltrombopag 50 mg p. o. daily	69	4.5 weeks	94%	48%	–	15 months^c^

*ITP* immune thrombocytopenia, *ORR* overall response rate defined as platelet count ≥30,000/µL, *CRR* complete response rate defined as platelet count ≥100,000/µL, *p.o*. per os, *s.c*. subcutaneously

^a^Difference in duration of response likely due to later onset of treatment with eltrombopag due to later drug availability

Furthermore, TRA were demonstrated to enhance health-related QoL as measured by ITP-PAQ and SF-36 [29, 30].

Safety of both agents is comparable with discontinuation of therapy due to adverse drug reactions in approximately 5–7% of patients [27, 30].

Besides hepatotoxicity associated with eltrombopag, suspected toxicities of TRA include thrombosis and bone marrow fibrosis. The incidence of thromboembolic complications, however, was demonstrated not to be significantly increased in a meta-analysis of 14 trials (921 patients) [31]. Whether TRA are associated with significant increase in reticulin and collagen fibrosis currently remains unclear.

Optimal treatment for patients in need of second-line therapy is still under debate. Some physicians favor splenectomy in the absence of a contraindication to surgery. Others underline missing evidence necessary for prioritization among available therapeutic options.

Typically, the choice of second-line therapy is discussed with the patients balancing the pros and cons of each option in the context of their individual comorbidities and preferences.

The only absolute contraindications of splenectomy are uncontrolled coagulopathy and severe cardiovascular disease prohibiting or severely compromising general anesthesia.

**Fig. 1:**
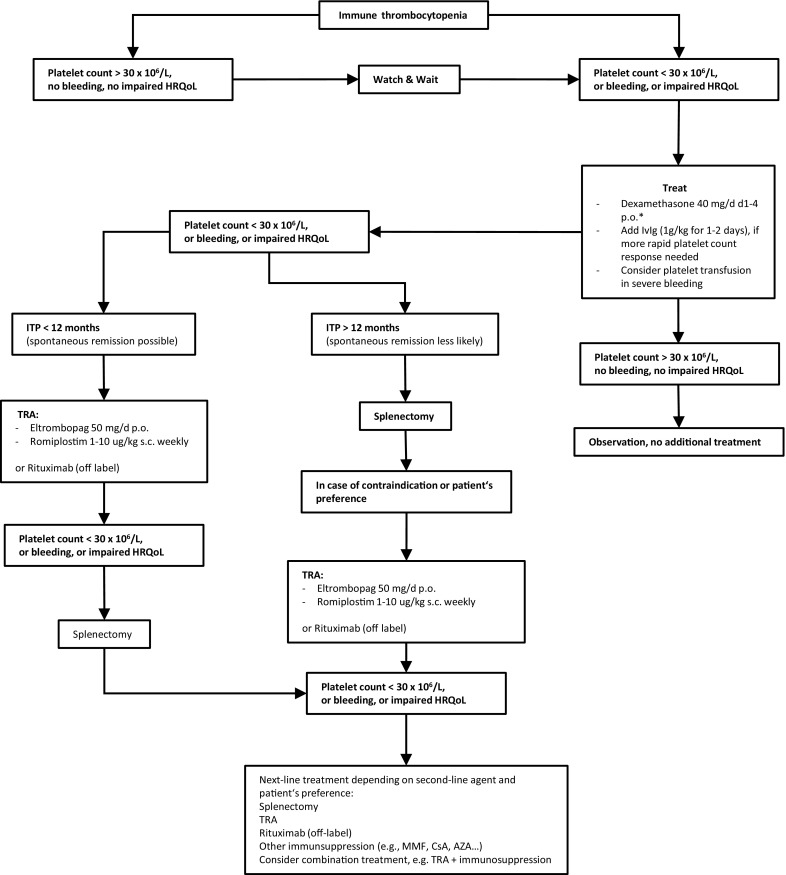
Treatment algorithm in adult immune thrombocytopenia. *ITP* immune thrombocytopenia, *IvIg* intravenous immunglobuline, *TRA* thrombopoietin receptor agonist, *MMF* mycophenolate mofetil, *CSA* cyclosporine A, *AZA* azathioprine, *HRQoL* health-releated quality of life, *p.o.* per os, *s.c.* subcutaneously, *d* day, * alternatively prednisolone 1‑2 mg/kg per day over several weeks with a 4‑6 week taper

Fig. 1 provides an overview of current management strategies in adult ITP.

## Conclusion

Most adults with ITP will eventually maintain a safe platelet count. The likelihood of long-term remission after standard corticosteroids, however, remains low even with intensified first-line treatment. Frequently, more than one line of therapy is required until a stable and durable response is achieved, mostly as a result of chronic treatment with TRA or splenectomy.

In this respect, it is crucial to investigate whether available drugs administered in novel combinations with corticosteroids, monoclonal antibodies or immunosuppressants can prevent relapse. Notably, adequate long-term follow-up including appropriate health-related QoL assessment is necessary to address this question in randomized controlled trials. Furthermore, it is critical to identify the subgroup of patients most at risk for developing chronic ITP and, thus, who would benefit from more intensive up-front treatment. This way, patients may be offered tailored therapies balancing between providing a safe and sustained platelet response and avoiding unnecessary treatment-related toxicities.
